# Association between dry eye disease and depression: An umbrella review

**DOI:** 10.3389/fpubh.2022.910608

**Published:** 2022-11-17

**Authors:** Ching-Yao Tsai, Zhu Liduzi Jiesisibieke, Tao-Hsin Tung

**Affiliations:** ^1^Department of Ophthalmology, Taipei City Hospital, Taipei, Taiwan; ^2^Institute of Public Health, National Yang Ming Chiao Tung University, Taipei, Taiwan; ^3^Department of Business Administration, Fu Jen Catholic University, Taipei, Taiwan; ^4^General Education Center, University of Taipei, Taipei, Taiwan; ^5^Li Ka Shing Faculty of Medicine, School of Public Health, The University of Hong Kong, Hong Kong, Hong Kong SAR, China; ^6^Taiwan Association of Health Industry Management and Development, Taipei, Taiwan

**Keywords:** dry eye disease, depression, umbrella review, meta-analysis, psychological status

## Abstract

**Purpose:**

This umbrella review aimed to summarize the available evidence on the association between dry eye disease and depression.

**Methods:**

We searched the Pubmed, Embase, and Cochrane databases using the search string “(Dry eye syndrome OR Keratoconjunctivitis sicca OR KCS OR Aqueous tear deficiency OR Sjogren syndrome) AND (depression OR depressive symptoms) AND (meta-analysis OR systematic review)” from inception to 20 July 2022. We considered all meta-analyses investigating the association between dry eye disease and depression.

**Results:**

After summarizing the included meta-analyses, it was concluded that dry eye disease is associated with depression. The symptoms of dry eye disease affect the daily lives of patients, thus affecting their mood. However, further evidence is required to confirm this association.

**Conclusion:**

This finding highlights the importance of psychological support for patients with dry eye disease. Future clinical studies should investigate the mechanism underlying the association between dry eye disease and depression.

**Systematic review registration:**

https://www.crd.york.ac.uk/PROSPERO/, identifier: CRD42022320619.

## Introduction

Dry eye disease, which affects the tear-air interface and ocular surface, is an increasing public health issue due to its influence on visual function and quality of life (1, 2). Dry eye disease is one of the most common ocular diseases worldwide, with an estimated prevalence of 11.59% (3). The usage of digital screen is associated with incident dry eye disease (4). The positive association between COVID-19 pandemic, prolonged screen times and the association between dry eye disease has already been proved (5–9). There are two subtypes of this disease, aqueous-deficient and evaporative. Keratoconjunctivitis sicca, Sjögren's syndrome and aqueous tear deficiency are all subgroups of the dry eye disease (10–12). Dry eye disease has been associated with many risk factors, including external adverse environmental factors (13). Air pollution, including higher ozone levels and particulate matter 2.5 pollution, was shown to cause ocular discomfort and induce dry eye disease (14). Seasonal changes also affect the prevalence of dry eye disease, which was found to be higher in the winter and summer (15).

Patients with dry eye disease often present with symptoms of eye discomfort, including stinging, burning, and pressure sensation, as well as sharp or throbbing pain (16). Consequently, many studies have found an association between dry eye disease and the patients' psychological status (17). A study conducted among older adults found that age-related reduction in tear production was associated with depression (18). A large population-based study identified a significant association between dry eye disease and depression in the general population (19). However, other studies have found no evidence of such significant association between Sjögren's syndrome and depression, and Sjögren's syndrome are one of the subgroups of dry eye disease (20, 21).

Given the inconsistency of prior findings and the fact that results vary according to gender, age, and other factors, the interaction between dry eye disease and depression remains unclear. Therefore, we performed an umbrella review of related meta-analyses to summarize the present knowledge on this topic and explore the possible explanations and effects.

## Methods

### Search strategy and eligibility criteria

In order to generate comprehensive results, we included only meta-analyses investigating the association between dry eye disease and depression. We searched the Pubmed, Cochrane, and Embase databases from inception to 20 July 2022, using the predetermined search string “(Dry eye syndrome OR Keratoconjunctivitis sicca OR KCS OR Aqueous tear deficiency OR Sjogren syndrome) AND (depression OR depressive symptoms) AND (meta-analysis OR systematic review).” There were no restrictions regarding the study language, subgroup of dry eye disease, or the age, gender, or race of participants. Only studies where depression or depressive symptoms developed subsequent to the diagnosis of dry eye disease were included. Studies that did not specify the cutoff threshold to detect depression were excluded. Studies investigating anxiety, cognitive impairment, or other psychological disorders were also excluded. This study was registered in the International Prospective Register of Systematic Reviews (PROSPERO ID: 320619).

### Data extraction

Based on the PRISMA 2020 guidelines, the search process could be done either by human or by automation tools (22). In our study, two of the authors (C-YT and ZJ) independently completed the study screening process. Any discrepancies were resolved through dialogue with the senior author (T-HT.). After duplicates were removed, the retrieved studies were screened for eligibility. We excluded the following studies: (1) did not perform a quantitative synthesis; (2) did not report adequate data. Eligible studies were evaluated based on the level of comparison, random-effects summary, I^2^ statistic. The following data were extracted: author, year, number of included participants, patients' disease, outcome measurement tools, I^2^ statistic, and number of study types, statistical significance, and largest study effect size. We have also calculated 95% prediction intervals.

### Assessment of methodological quality

Two of the authors (C-YT and ZJ) independently followed the AMSTAR-2 (a measurement tool to assess systematic reviews) guideline to assess the methodological quality of the included meta-analyses. The disagreements were resolved through a discussion with a senior author (T-HT). AMSTAR-2 is often used in umbrella review as it covers various aspects (23, 24). This guideline includes 16 aspects that systematically grade evidence-based medical papers (24, 25). The shortcomings in each of the aspect would result in overall quality (23). Because a high overall score may obscure some of the serious limitations of the included studies and they may be regarded as high-quality studies, a sum score for each part is not provided (26) (Table 1). The AMSTAR-2 is regarded as a reliable and valid method for evaluating the quality of systematic reviews and meta-analyses of interventional and observational research (24, 25, 32). Compared with the Risk of Bias In Non-randomized Studies-of Interventions tool, which has been commonly used in reviews, the AMSTAR-2 evaluates the determination of the study design for inclusion, reasons for exclusion of studies, sources of funding of the primary studies, and reviewers' conflict of interest (33).

**Table 1:** Characteristics of the included studies.

**No**.	**Author, year, country**	**Number of included participants**	**Patients' disease**	**Measurement of outcome**	**I^2^**	**Statistical significance**	**Summary effect size**	**95% prediction interval**	**Largest study effect size**	**Excess** **significance bias**	**Selection** **as most** **comprehensive**	**RCTs** **included**	**Prospective** **studies included**	**Retrospective** **studies included**	**Study quality** **(AMSTAR) rating**
1	Al-Ezzi et al. (27) 2016, UK	313 (exposure [SSDE]: 164; control: 149)	Sjogren's syndrome dry eye	HADS	57%	*p* < 0.0001	MD: 0.79 (95% CI:0.43,1.15)	(-7.585, 14.424)	0.37 (-0.01,0.74)	No		0	0	4	Low
2	Wan et al. (28), 2016, Hong Kong (China)	2,978,844 (Exposure [Non-SSDE]: 482,383; control: 2,482,982; [SSDE], exposure: 2,654; control: 10,825)	SSDE & non-SSDE	SCL-9; Zung; HADS; CES-D; ICD-9; Beck; PHQ-9;	99%; 72%	*p* < 0.0001; *p* < 0.00001	Non-SSDE (OR): 2.24 (95% CI: 1.50, 3.33); SSDE (OR): 4.25 (95% CI: 2.67, 6.78)	(0.821, 5.859) (1.095,16.794)	0.21 (0.07,0.34); 0.64 (0.28,1.00)	No; No	√	0	0	13	Low
3	Zheng et al. (29), 2017, China	6,589 (event: 1,502, total: 6,589)	Dry eye disease	HADS; CES-D; PHQ-9; Zung; GADS	96.5%	*p* = 0.000	Prevalence: 0.25 (0.20, 0.30)	(0,0.513)	0.05 (0.04,0.07)	No	√	0	0	5	Low
4	Cui et al. (30), 2017, China	1,441 (exposure [with SSDE]: 604; control (without SSDE): 837)	Sjogren's syndrome dry eye	HADS; CES-D; Beck; Zung; PHQ-9	30%	*p* < 0.00001	OR: 5.36 (95% CI: 4.05, 7.09)	(1.533, 21.115)	4.32 (3.06,6.11)	No		0	1	11	Moderate
5	Basilious et al. (31), 2021, Canada	17,694 (exposure [with dry eye disease]: 2,201; control [without dry eye disease]: 15,493)	Dry eye disease	PHQ-9; Interview; DASS; Clinical diagnosis; HADS; Beck; MADRS; QIDS; CES-D;	73.3%	*p* = 0.001	OR: 1.81 (1.61, 2.02)	(0.907,7.933)	1.58 (1.39,1.79)	No	√	0	0	12	Low

Beck, Beck depression inventory; CES-D, The Centers for Epidemiologic Studies Depression Scale; HADS, Hospital Anxiety and Depression Scale; ICD-9, International Classification of Diseases coding; PHQ-9, Patient Health Questionnaire; SCL-9OR, symptom checklist 90R; Zung, Zung-Self rating depression or anxiety scale; DASS, Depression Anxiety Stress Scales; MADRS, Montgomery-Asberg Depression Scale; QIDS-SR16, Quick Inventory of Depressive Symptomatology - Japanese version; GADS, Goldberg Anxiety and Depression Scale; SSDE, Sjogren's syndrome dry eye.

Largest study significance: ES of the largest study (smallest error) in each meta-analysis.

### Assessment of epidemiological credibility

We reanalyze all the included meta-analysis. High epidemiological credibility means highest evidence and no hints of major heterogeneity or bias (34). We further classified the included studies into the following types (35):

1. Persuasive: statistical significance per the random-effects model of *p* < 0.000001, more than 1,000 cases, no high heterogeneity among the selected studies (I^2^ < 50%), 95% CI (excluding the null value), and no evidence of small-study effects and significant bias;
2. Highly recommended: statistical significance of *p* < 0.000001, more than 1,000 cases, and most studies indicating a significant effect;
3. Recommended: more than 1,000 cases and significant effects at *p* < 0.001;
4. Weak evidence: nominally significant associations (*p* < 0.05); and
5. Poor evidence: obtained from samples with <1,000 cases.

### Assessment of small-study effects and excess significance bias

Small study effects describe a phenomenon that smaller studies sometimes showed larger treatment effects than larger studies (36). This is often caused by publication bias, and the *p*-value of Egger's test below 0.10 if small study effects exist (37). To better understand the characteristics of the included studies, we also evaluated the excess significant bias of each included study. Excess significant bias is based on the observed (O) number of studies (studies with significant result: *p* < 0.05) and the expected (E) number of the studies (38). Expected number (E) of the studies were calculated through the sum of the statistical power estimates for each included component study. The power of each included study was calculated using a non-central t distribution (37–39). Excess statistical significance for each meta-analyses was determined at two-sided *p* < 0.10 with O > E as previously proposed (37–39).

## Results

### Study characteristics

**Figure 1:**
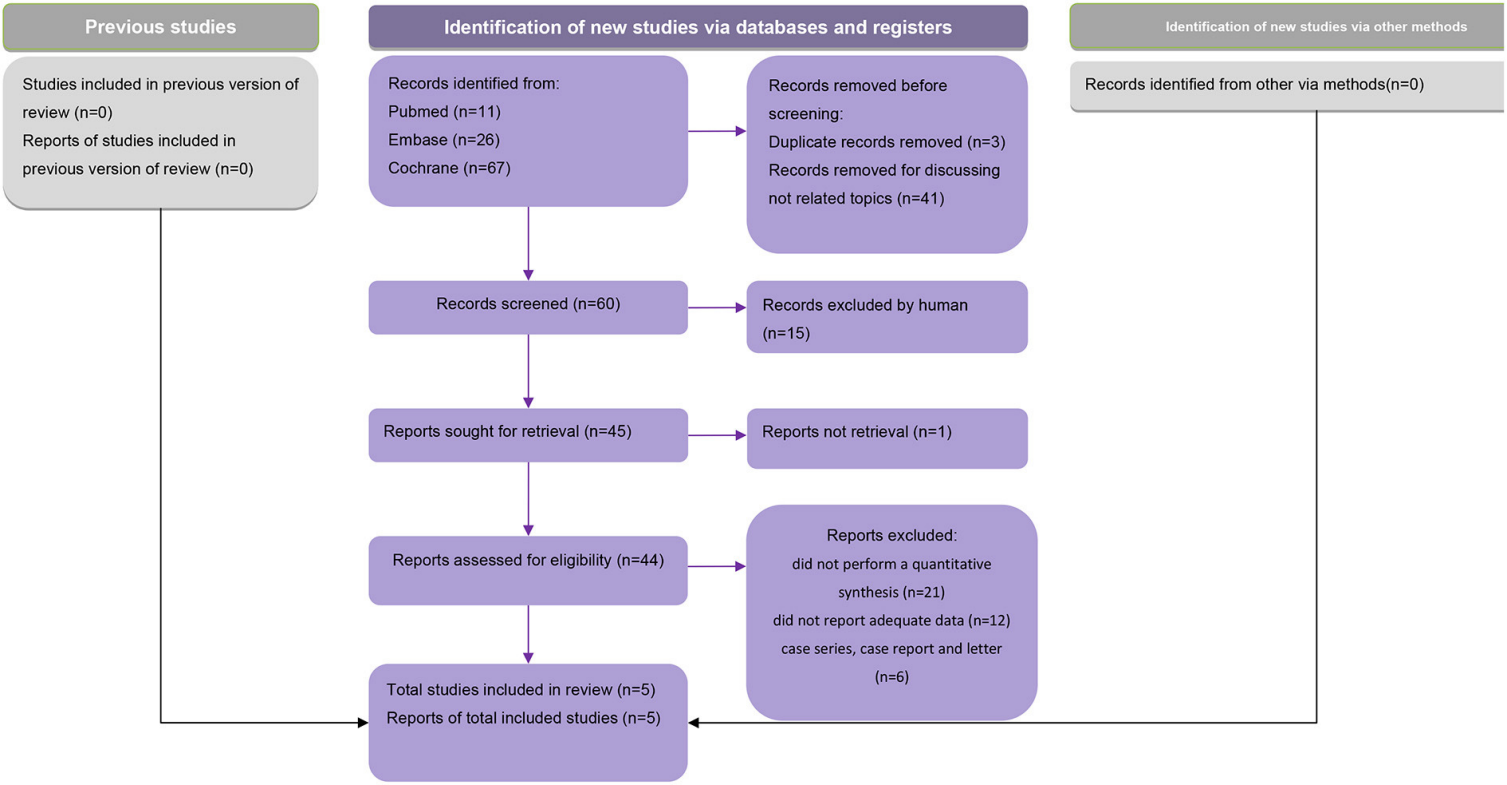
PRISMA flow chart.

After excluding duplicates, a total of 95 meta-analyses were screened for eligibility, of which five that met the eligibility criteria were included in our analysis (Figure 1). These five studies were published between 2016 and 2021. Two of the studies defined the outcome as primary Sjogren's syndrome-related dry eye disease (27, 30), other two simply as dry eye disease (29, 31), and in one study, a subgroup analysis was performed and both Sjogren's syndrome- and non-Sjogren's syndrome-related dry eye disease were included in the outcomes (28). Based on the criteria and the characteristics of each included study, we classified our included studies into several groups. The study conducted by Cui et al. (30), Zheng et al. (29), and Wan et al. (28) could be classified as “recommended.” While the study by Al-Ezzi et al. (27) and Basilious et al. (31) were considered as “weak evidence.”

### Outcome analysis

Al-Ezzi et al. (27) included five studies comprising 313 female patients and found that primary Sjogren's syndrome-related dry eye disease had an adverse effect on the psychological status of females. Cui et al. (30) found that patients with primary Sjogren's syndrome-related dry eye disease had higher prevalence and greater severity of depression compared with those in the control groups. Wan et al. (28) concluded that patients with either Sjogren's syndrome- or non-Sjogren's syndrome-related dry eye disease were more likely to develop depression. Zheng et al. (29) included 28 studies and found a significant association between dry eye disease and depression. Basilious et al. (31) concluded that depression was associated with dry eye disease symptoms but not signs.

### Outcome measurement

The outcome measurement tools varied across studies and included the Hospital Anxiety and Depression Scale (HADS), Zung Self-rating depression or anxiety scale (Zung), Centers for Epidemiologic Studies Depression Scale (CES-D), and other. Although different tools were used, most of the included studies described the measurement in detail.

### Publication bias

The publication bias assessment also differed among the five included studies. Al-Ezzi et al. (27) and Cui et al. (30) did not use funnel plots to assess the publication bias because the number of included studies in their meta-analyses was <10. Wan et al. (28) did not assess the publication bias in detail. Zheng et al. (29) revealed no significant publication bias based on Begg's test. Basilious et al. (31) used funnel plots to assess the publication bias, and although they found asymmetry, they did not regard it as a result of publication bias.

### Small study effects and excess significance bias

In this umbrella review, none of the included studies provided the results of Egger's test. Therefore, we could not evaluate the small-study effects. The results of excess significance bias were shown in Table 1.

### Residual confounding

All the included studies were observational studies; therefore, we cannot conclude the causal relationship between dry eye disease and depression. We acknowledge there may be some residual confounding or other biases when studying the association between dry eye disease and depression, and the existence of residual confounding or other biases may lead to overestimation or underestimation (40) and restricted causal interferes (41).

## Discussion

All studies included in this umbrella review found a significant association between dry eye disease symptoms and depression, although the results varied across different characteristics of patients. Exploring this association may have important implications for the treatment of patients with dry eye disease.

### Clinical implications

The prevalence of dry eye disease among people over 40 can be 75% (42). According to the data published by the World Health Organization, depression is estimated to affect 5.0% of adults globally (43). This review confirmed the previously reported association between dry eye disease and depression; however, the mechanism underlying this interaction remains to be explored.

The association between dry eye disease and depression tends to be bidirectional, i.e., depression can be both the cause and effect of dry eye disease (31). A prior study that demonstrated that patients with depression were likely to develop dry eye disease noted some important similarities between the pathophysiological mechanisms of the two conditions. Namely, the levels of the inflammatory cytokines interleukin-1, interleukin-6, and tumor necrosis factor (13) are increased both in depression and dry eye disease (44). These two conditions also have some risk factors in common, including female sex (45, 46), smart phone use (47, 48), gout (49, 50), and menopause (51, 52) and some other factors. Vehof et al. (45) examined the dry eye disease in a British female cohort, and discussed that hormone changes, lower pain thresholds in female may be possible reasons of the prevalence of depression among female dry eye disease patients. Zheng et al. (29) found that aged patients may easily to feel helpless and other negative feelings.

The coexistence of dry eye disease and depression was found to be associated with a series of unpleasant outcomes, including sleep disorder (53), suicide risk (54), reduced work productivity (55), and lower rates of treatment compliance (56). Furthermore, the eye discomfort affects these patients' personal feelings (57), and even their daily activities, such as reading, driving, watching television, and computer use (17, 57–59). Patients with dry eye disease often feel sad and pessimistic, and are also likely to experience cognitive disorders and pain (29). There is also a significant association between dry eye disease and anxiety, suggesting that patients with dry eye disease are more anxious and depressed compared with a healthy population (60). The biological mechanism between dry eye disease and depression is of interdisciplinary field. Some of the studies indicated that dry eye disease affects the quality of life, and then affects central nervous system sensitization (53). Another study investigated the single nucleotide polymorphisms (SNPs) in the genes in patients with dry eye disease, and they found that *Val66Met, Fokl*, and *Apal* was associated with dry eye disease. Furthermore, this association was affected by depression status (61). However, in the mechanism, which occurs first in dry eye and depression is not that clear.

The finding that depression and dry eye disease are closely related can contribute to the treatment of dry eye disease, as well as to the further investigation of the mechanism underlying the relationship between dry eye disease and psychological disorders. Based on the above, patients with dry eye disease should be provided psychological support.

### Strengths and limitations

To the best of our knowledge, this is the first umbrella review to summarize the evidence on the association between dry eye disease and depression. Most of the included studies used objective measurement tools both for depression and dry eye disease, suggesting reliable results, which has high clinical value in real scenario. In view of the association between usage of digital screen and dry disease (4) and the fact of increasing screen-time around the world, we believe this study is of great interest of the public.

Nonetheless, this study also had several limitations. First, since the results and effect size varied among the included studies, we could not calculate a pooled result. Second, most of the included studies were retrospective. Hence, future prospective studies are needed to investigate the causality between dry eye disease and depression. Third, the differences among the studies in the measurement tools for depression, sample size, and other factors resulted in statistical heterogeneity; thus, the results should be interpreted with caution. Fourth, further studies should summarize the evidence on the association between the severity of dry eye disease and that of depression. Fifth, we only explored the association between dry eye and depression, however, we acknowledged that the association between other eye disease or their treatments [such as retinopathy (62, 63), cataract surgery (64), and glaucoma (65, 66)] and depression has already been proved in previously published meta-analysis. Finally, the high-quality meta-analysis investigating the association between dry eye disease and depression are lacking currently. Therefore, more high-quality meta-analysis should be conducted to include all eye disease including dry eye disease in the future.

## Conclusion

This review confirmed the association between dry eye disease and patients' psychological status, emphasizing the importance of psychological support and guidance for these patients. Future clinical studies are needed to explore the mechanism underlying this relationship.

## Data availability statement

The original contributions presented in the study are included in the article/supplementary material, further inquiries can be directed to the corresponding author.

## Author contributions

C-YT, ZJ, and T-HT conducted the study and drafted the manuscript. C-YT and ZJ participated in the design of the study and performed statistical analyses. T-HT conceived the study and participated in its design and coordination. All authors read and approved the final manuscript. All authors contributed to the article and approved the submitted version.

## Conflict of interest

The authors declare that the research was conducted in the absence of any commercial or financial relationships that could be construed as a potential conflict of interest.

## Publisher's note

All claims expressed in this article are solely those of the authors and do not necessarily represent those of their affiliated organizations, or those of the publisher, the editors and the reviewers. Any product that may be evaluated in this article, or claim that may be made by its manufacturer, is not guaranteed or endorsed by the publisher.

## References

[1] Verjee MA Brissette AR Starr CE. Dry eye disease: early recognition with guidance on management and treatment for primary care family physicians. Ophthalmol Ther. (2020) 9:877–88. 10.1007/s40123-020-00308-z 33090327 PMC7708574

[2] Aragona P Giannaccare G Mencucci R Rubino P Cantera E Rolando M. Modern approach to the treatment of dry eye, a complex multifactorial disease: a P. ICASSO board review. Br J Ophthalmol. (2021) 105:446–53. 10.1136/bjophthalmol-2019-315747 32703782 PMC8005804

[3] Papas EB. The global prevalence of dry eye disease: aA Bayesian view. Ophthalmic Physiol Opt. (2021) 41:1254–66. 10.1111/opo.12888 34545606

[4] Al-Mohtaseb Z Schachter S Shen Lee B Garlich J Trattler W. The relationship between dry eye disease and digital screen use. Clin Ophthalmol. (2021) 15:3811–20. 10.2147/OPTH.S321591 34531649 PMC8439964

[5] Elhusseiny AM Eleiwa TK Yacoub MS George J ElSheikh RH Haseeb A . Relationship between screen time and dry eye symptoms in pediatric population during the COVID-19 pandemic. Ocul Surf. (2021) 22:117–9. 10.1016/j.jtos.2021.08.002 34363976 PMC9760210

[6] Pandey SK Sharma V. Mask-associated dry eye disease and dry eye due to prolonged screen time: Are we heading towards a new dry eye epidemic during the COVID-19 era? Indian J Ophthalmol. (2021) 69:448. 10.4103/ijo.IJO_3250_20 33380621 PMC7933894

[7] Prescott CR. Increased screen time and dry eye: another complication of COVID-19. Eye Contact Lens. (2021) 47:433. 10.1097/ICL.0000000000000820 34310487 PMC8294656

[8] Tangmonkongvoragul C Chokesuwattanaskul S Khankaeo C Punyasevee R Nakkara L Moolsan S . Prevalence of symptomatic dry eye disease with associated risk factors among medical students at Chiang Mai University due to increased screen time and stress during COVID-19 pandemic. PLoS ONE. (2022) 17:e0265733. 10.1371/journal.pone.0265733 35320310 PMC8942203

[9] Neti N Prabhasawat P Chirapapaisan C Ngowyutagon P. Provocation of dry eye disease symptoms during COVID-19 lockdown. Sci Rep. (2021) 11:1–9. 10.1038/s41598-021-03887-4 34952901 PMC8709849

[10] Lemp MA Foulks GN. The definition and classification of dry eye disease. Ocul Surf. (2007) 5:75–92. 10.1016/S1542-0124(12)70081-2 17508116

[11] Chang S-W Wu W-L. Association between dry eye parameters depends on tear components. J Clin Med. (2022) 11:3056. 10.3390/jcm11113056 35683444 PMC9181409

[12] Da Cunha E Mariette X Desmoulins F Bergé E Nocturne G Benmalek A . Associations between ocular and extra-ocular assessment in primary Sjögren's syndrome. Joint Bone Spine. (2022) 89:105426. 10.1016/j.jbspin.2022.105426 35716880

[13] Calonge M Pinto-Fraga J González-García MJ Enríquez-de-Salamanca A López-de la Rosa A Fernández I . Effects of the external environment on dry eye disease. Int Ophthalmol Clinics. (2017) 57:23–40. 10.1097/IIO.0000000000000168 28282312

[14] Kim Y Choi Y-H Kim MK Paik HJ Kim DH. Different adverse effects of air pollutants on dry eye disease: Ozone, PM2. 5, and PM10. Environ Pollut. (2020) 265:115039. 10.1016/j.envpol.2020.115039 32806456

[15] van Setten G Labetoulle M Baudouin C Rolando M. Evidence of seasonality and effects of psychrometry in dry eye disease. Acta Ophthalmologica. (2016) 94:499–506. 10.1111/aos.12985 27105776

[16] Guo LW AKPEK E. The negative effects of dry eye disease on quality of life and visual function. Turk J Med Sci. (2020) 50:1611–5. 10.3906/sag-2002-143 32283910 PMC7672346

[17] Uchino M Schaumberg DA. Dry eye disease: impact on quality of life and vision. Curr Ophthalmol Rep. (2013) 1:51–7. 10.1007/s40135-013-0009-1 23710423 PMC3660735

[18] Kim KW Han SB Han ER Woo SJ Lee JJ Yoon JC . Association between depression and dry eye disease in an elderly population. Invest Ophthalmol Vis Sci. (2011) 52:7954–8. 10.1167/iovs.11-8050 21896858

[19] Van Der Vaart R Weaver MA Lefebvre C Davis RM. The association between dry eye disease and depression and anxiety in a large population-based study. Am J Ophthalmol. (2015) 159:470–4. 10.1016/j.ajo.2014.11.028 25461298 PMC4329250

[20] Bongi SM Del Rosso A Orlandi M Matucci-Cerinic M. Gynaecological symptoms and sexual disability in women with primary Sjögren's syndrome and sicca syndrome. Clin Exp Rheumatol. (2013) 31:683–90. 23710558

[21] Theander L Strömbeck B Mandl T Theander E. Sleepiness or fatigue? Can we detect treatable causes of tiredness in primary Sjögren's syndrome? Rheumatology. (2010) 49:1177–83. 10.1093/rheumatology/keq023 20308122

[22] Page MJ Moher D Bossuyt PM Boutron I Hoffmann TC Mulrow CD . PRISMA 2020 explanation and elaboration: updated guidance and exemplars for reporting systematic reviews. BMJ. (2021) 372:n160. 10.1136/bmj.n160 33781993 PMC8005925

[23] Okoth K Chandan JS Marshall T Thangaratinam S Thomas GN Nirantharakumar K . Association between the reproductive health of young women and cardiovascular disease in later life: umbrella review. BMJ. (2020) 371:m3502. 10.1136/bmj.m3502 33028606 PMC7537472

[24] Poole R Kennedy OJ Roderick P Fallowfield JA Hayes PC Parkes J. Coffee consumption and health: umbrella review of meta-analyses of multiple health outcomes. BMJ. (2017) 359:j5024. 10.1136/bmj.j5024 29167102 PMC5696634

[25] Shea BJ Hamel C Wells GA Bouter LM Kristjansson E Grimshaw J . AMSTAR is a reliable and valid measurement tool to assess the methodological quality of systematic reviews. J Clin Epidemiol. (2009) 62:1013–20. 10.1016/j.jclinepi.2008.10.009 19230606

[26] Shea BJ Reeves BC Wells G Thuku M Hamel C Moran J . AMSTAR 2: a critical appraisal tool for systematic reviews that include randomised or non-randomised studies of healthcare interventions, or both. BMJ. (2017) 358:j4008. 10.1136/bmj.j4008 28935701 PMC5833365

[27] Al-Ezzi MY Pathak N Tappuni AR Khan KS. Primary Sjögren's syndrome impact on smell, taste, sexuality and quality of life in female patients: a systematic review and meta-analysis. Mod Rheumatol. (2017) 27:623–9. 10.1080/14397595.2016.1249538 27760487

[28] Wan KH Chen LJ Young AL. Depression and anxiety in dry eye disease: a systematic review and meta-analysis. Eye. (2016) 30:1558–67. 10.1038/eye.2016.186 27518547 PMC5177754

[29] Zheng Y Wu X Lin X Lin H. the prevalence of depression and depressive symptoms among eye disease patients: a systematic review and meta-analysis. Sci Rep. (2017) 7:46453. 10.1038/srep46453 28401923 PMC5388862

[30] Cui Y Li L Yin R Zhao Q Chen S Zhang Q . Depression in primary Sjögren's syndrome: a systematic review and meta-analysis. Psychol Health Med. (2018) 23:198–209. 10.1080/13548506.2017.1339895 28621153

[31] Basilious A Xu CY Malvankar-Mehta MS. Dry eye disease and psychiatric disorders: a systematic review and meta-analysis. Eur J Ophthalmol. 2021:11206721211060963. 10.1177/11206721211060963 34935549 PMC9297048

[32] Pieper D Mathes T Eikermann M. Can AMSTAR also be applied to systematic reviews of non-randomized studies? BMC Res Notes. (2014) 7:609. 10.1186/1756-0500-7-609 25193554 PMC4167129

[33] Swierz MJ Storman D Zajac J Koperny M Weglarz P Staskiewicz W . Similarities, reliability and gaps in assessing the quality of conduct of systematic reviews using AMSTAR-2 and ROBIS: systematic survey of nutrition reviews. BMC Med Res Methodol. (2021) 21:261. 10.1186/s12874-021-01457-w 34837960 PMC8627612

[34] Bellou V Belbasis L Tzoulaki I Evangelou E. Risk factors for type 2 diabetes mellitus: An exposure-wide umbrella review of meta-analyses. PLoS ONE. (2018) 13:e0194127. 10.1371/journal.pone.0194127 29558518 PMC5860745

[35] Gu D-T Tung T-H Jiesisibieke ZL Chien C-W Liu W-Y. Safety of cinnamon: an umbrella review of meta-analyses and systematic reviews of randomized clinical trials. Front Pharmacol. (2021) 12:790901. 10.3389/fphar.2021.790901 35115937 PMC8804376

[36] Schwarzer G Carpenter JR Rücker G. Small-study effects in meta-analysis. In: Meta-Analysis with R. Use R. Cham: Springer (2015). 10.1007/978-3-319-21416-0_5

[37] Bellou V Belbasis L Tzoulaki I Evangelou E Ioannidis JPA. Environmental risk factors and Parkinson's disease: an umbrella review of meta-analyses. Parkinsonism Relat Disord. (2016) 23:1–9. 10.1016/j.parkreldis.2015.12.008 26739246

[38] Ioannidis JPA Trikalinos TA. An exploratory test for an excess of significant findings. Clin Trials. (2007) 4:245–53. 10.1177/1740774507079441 17715249

[39] Sun H Gong T-T Xia Y Wen Z-Y Zhao L-G Zhao Y-H . Diet and ovarian cancer risk: an umbrella review of systematic reviews and meta-analyses of cohort studies. Clin Nutr. (2021) 40:1682–90. 10.1016/j.clnu.2020.11.032 33308841

[40] Raglan O Kalliala I Markozannes G Cividini S Gunter MJ Nautiyal J . Risk factors for endometrial cancer: an umbrella review of the literature. Int J Cancer. (2019) 145:1719–30. 10.1002/ijc.31961 30387875

[41] Han MA Zeraatkar D Guyatt GH Vernooij RWM El Dib R Zhang Y . Reduction of red and processed meat intake and cancer mortality and incidence: a systematic review and meta-analysis of cohort studies. Ann Intern Med. (2019) 171:711–20. 10.7326/M19-0699 31569214

[42] Rouen PA White ML. Dry eye disease: prevalence, assessment, and management. Home Healthcare Now. (2018) 36:74–83. 10.1097/NHH.0000000000000652 29498987

[43] Evaluation IoHMa. Global Health Data Exchange (GHDx). Available online at: https://vizhub.healthdata.org/gbd-results/?params=gbd-api-2019-permalink/d780dffbe8a381b25e1416884959e88b

[44] Tiskaoglu NS Yazici A Karlidere T Sari E Oguz EY Musaoglu M . Dry eye disease in patients with newly diagnosed depressive disorder. Curr Eye Res. (2017) 42:672–6. 10.1080/02713683.2016.1236966 27870590

[45] Vehof J Kozareva D Hysi PG Hammond CJ. Prevalence and risk factors of dry eye disease in a British female cohort. Br J Ophthalmol. (2014) 98:1712–7. 10.1136/bjophthalmol-2014-305201 25185440

[46] Albert PR. Why is depression more prevalent in women? J Psychiatry Neurosci. (2015) 40:219–21. 10.1503/jpn.150205 26107348 PMC4478054

[47] Elhai JD Dvorak RD Levine JC Hall BJ. Problematic smartphone use: a conceptual overview and systematic review of relations with anxiety and depression psychopathology. J Affect Disord. (2017) 207:251–9. 10.1016/j.jad.2016.08.030 27736736

[48] Moon JH Kim KW Moon NJ. Smartphone use is a risk factor for pediatric dry eye disease according to region and age: a case control study. BMC Ophthalmol. (2016) 16:1–7. 10.1186/s12886-016-0364-4 27788672 PMC5084437

[49] Lin S Zhang H Ma A. Association of gout and depression: a systematic review and meta-analysis. Int J Geriatr Psychiatry. (2018) 33:441–8. 10.1002/gps.4789 28921661

[50] Lee C-Y Chen H-C Sun C-C Lin H-Y Lu K-H Huang J-Y . Gout as a risk factor for dry eye disease: a population-based cohort study. J Clin Med. (2019) 8:62. 10.3390/jcm8010062 30634389 PMC6352280

[51] Freeman EW. Associations of depression with the transition to menopause. Menopause. (2010) 17:823–7. 10.1097/gme.0b013e3181db9f8b 20531231

[52] Versura P Campos EC. Menopause and dry eye. A possible relationship. Gynecol Endocrinol. (2005) 20:289–98. 10.1080/09513590400027257 16019376

[53] JM Weatherby T Ram Vasant Raman V Agius M. Depression and dry eye disease: a need for an interdisciplinary approach? Psychiatr Danub. (2019) 31:619–21. 31488802

[54] Um S-B Yeom H Kim NH Kim HC Lee HK Suh I. Association between dry eye symptoms and suicidal ideation in a Korean adult population. PLoS ONE. (2018) 13:e0199131. 10.1371/journal.pone.0199131 29924835 PMC6010274

[55] Uchino M Uchino Y Dogru M Kawashima M Yokoi N Komuro A . Dry eye disease and work productivity loss in visual display users: the Osaka study. Am J Ophthalmol. (2014) 157:294–300. 10.1016/j.ajo.2013.10.014 24184225

[56] Jamilloux Y Sarabi M Kérever S Boussely N Le Sidaner A Valgueblasse V . Adherence to online monitoring of patient-reported outcomes by patients with chronic inflammatory diseases: a feasibility study. Lupus. (2015) 24:1429–36. 10.1177/0961203315585814 25966927

[57] Tong L Waduthantri S Wong T Saw S Wang J Rosman M . Impact of symptomatic dry eye on vision-related daily activities: the Singapore Malay Eye Study. Eye. (2010) 24:1486–91. 10.1038/eye.2010.67 20489740

[58] Deschamps N Ricaud X Rabut G Labbé A Baudouin C Denoyer A. The impact of dry eye disease on visual performance while driving. Am J Ophthalmol. (2013) 156:184–9. e3. 10.1016/j.ajo.2013.02.019 23706501

[59] Miljanović B Dana R Sullivan DA Schaumberg DA. Impact of dry eye syndrome on vision-related quality of life. Am J Ophthalmol. (2007) 143:409–15. e2. 10.1016/j.ajo.2006.11.060 17317388 PMC1847608

[60] Szakáts I Sebestyén M Németh J Birkás E Purebl G. The role of health anxiety and depressive symptoms in dry eye disease. Curr Eye Res. (2016) 41:1044–9. 10.3109/02713683.2015.1088955 26642862

[61] Hallak JA Tibrewal S Mohindra N Gao X Jain S. Single nucleotide polymorphisms in the BDNF, VDR, and DNASE 1 genes in dry eye disease patients: a case-control study. Invest Ophthalmol Vis Sci. (2015) 56:5990–6. 10.1167/iovs.15-17036 26393465 PMC5102496

[62] Zou Y You W Wang J Wang F Tian Z Lu J . Depression and retinopathy in patients with type 2 diabetes mellitus: a meta-analysis. Psychosom Med. (2021) 83:239–46. 10.1097/PSY.0000000000000924 33657086

[63] Li S Liu H Zhu X. The effect of psychotherapy on anxiety, depression, and quality of life in patients with diabetic retinopathy: a protocol for systematic review and network meta-analysis. Medicine. (2021) 100:e28386. 10.1097/MD.0000000000028386 34941170 PMC8702293

[64] Pellegrini M Bernabei F Schiavi C Giannaccare G. Impact of cataract surgery on depression and cognitive function: systematic review and meta-analysis. Clin Exp Ophthalmol. (2020) 48:593–601. 10.1111/ceo.13754 32220125

[65] Liu J Cao L Yang G Zhou R. Effects of non-pharmacological interventions on anxiety, depression, and sleep quality in patients with postoperative glaucoma: a protocol for systematic review and network meta-analysis. Medicine. (2021) 100:e27090. 10.1097/MD.0000000000027090 34477144 PMC8415987

[66] Groff ML Choi B Lin T McLlraith I Hutnik C Malvankar-Mehta MS. Anxiety, depression, and sleep-related outcomes of glaucoma patients: systematic review and meta-analysis. Can J Ophthalmol. (2022). 10.1016/j.jcjo.2022.02.010 35305959

